# The Core Binding Factor CBF Negatively Regulates Skeletal Muscle Terminal Differentiation

**DOI:** 10.1371/journal.pone.0009425

**Published:** 2010-02-25

**Authors:** Ophélie Philipot, Véronique Joliot, Ouardia Ait-Mohamed, Céline Pellentz, Philippe Robin, Lauriane Fritsch, Slimane Ait-Si-Ali

**Affiliations:** Institut André Lwoff, FRE2944, CNRS and Université Paris-Sud, Villejuif, France; Ludwig-Maximilians-Universität München, Germany

## Abstract

**Background:**

Core Binding Factor or CBF is a transcription factor composed of two subunits, Runx1/AML-1 and CBF beta or CBFβ. CBF was originally described as a regulator of hematopoiesis.

**Methodology/Principal Findings:**

Here we show that CBF is involved in the control of skeletal muscle terminal differentiation. Indeed, downregulation of either Runx1 or CBFβ protein level accelerates cell cycle exit and muscle terminal differentiation. Conversely, overexpression of CBFβ in myoblasts slows terminal differentiation. CBF interacts directly with the master myogenic transcription factor MyoD, preferentially in proliferating myoblasts, *via* Runx1 subunit. In addition, we show a preferential recruitment of Runx1 protein to MyoD target genes in proliferating myoblasts. The MyoD/CBF complex contains several chromatin modifying enzymes that inhibits MyoD activity, such as HDACs, Suv39h1 and HP1β. When overexpressed, CBFβ induced an inhibition of activating histone modification marks concomitant with an increase in repressive modifications at MyoD target promoters.

**Conclusions/Significance:**

Taken together, our data show a new role for Runx1/CBFβ in the control of the proliferation/differentiation in skeletal myoblasts.

## Introduction

Runx1 (for Runt-related transcription factor 1, also known as AML1 for Acute Myeloid Leukemia 1, CBFA2 or PEPB2αB) belongs to a family of highly homologous heterodimeric transcription factors named Core Binding Factors or CBF (reviewed in: [1]). In addition to the Runx1 subunit which binds DNA directly, CBF is composed of a non-DNA-binding subunit named CBFbeta (CBFβ) [2]. Runx1 binds better DNA in the presence of CBFβ. *Runx1* was originally identified at a breakpoint on human chromosome 21 in the t(8;21) translocation, known as the most common target of chromosomal translocations in human leukemia [3], [4]. Genetic studies showed that Runx1 is essential in the developing murine embryo for definitive hematopoiesis of all lineages [5], [6].

There is now strong evidence that Runx proteins are also important for differentiation of multiple cell types, including osteoblasts [7], neurons [8], [9], hematopoietic cells of all lineages [5], [6], [10] and skin epidermis and hair follicle stem cells [11], [12]. Runx1 is also involved in promoting senescence in primary mouse fibroblasts [13], and in cell cycle regulation [14]–[16].

Runx proteins have the potential to either activate or repress transcription in a context dependent manner. Runx1 seems to promote proliferation in progenitor cells, whereas in differentiating cells it cooperates with tissue-specific transcription factors to regulate tissue-specific gene expression. For example, Runx1 cooperates with C/EBPα and C/EBPβ to regulate hematopoiesis and osteogenesis, respectively [17], [18]. The dual role of Runx1 in regulating proliferation and differentiation could depend on differential interactions with protein partners, specific for each stage of cell development. The molecular mechanisms underlying such a switch in Runx1 function remain however to be deciphered.

Runx1 and CBFβ have also been linked to skeletal muscle differentiation [19]–[21], and prevention of muscle wasting [20]. In skeletal muscle, proliferation and differentiation are mutually exclusive. Indeed, skeletal muscle terminal differentiation begins with an irreversible withdrawal from the cell cycle, followed by muscle-specific marker expression [22]. Irreversible cell cycle exit involves a definitive silencing of proliferation-associated genes (reviewed in [23] and references therein). Terminal muscle differentiation is orchestrated by myogenic bHLH transcription factors, such as MyoD and Myf5, two master myogenic determination factors. MyoD is expressed in proliferating myoblasts, but is unable to activate its target genes even when bound to their promoters [24], [25]. MyoD therefore may have a repressive role at its target genes prior to initiating chromatin remodeling in differentiating cells [24], [26], [27]. In proliferating myoblasts, MyoD is associated with histone deacetylases (HDACs), the histone methyltransferase Suv39h1 and heterochromatin protein HP1, and might actively inhibit expression of its target genes by inducing a local repressive chromatin structure [24], [28], [29].

Here we show that CBF associates with MyoD preferentially in proliferating myoblasts, and knockdown of Runx1 or CBFβ accelerates cell cycle exit and terminal differentiation. Conversely, overexpression of CBF slows cell cycle exit and delays muscle differentiation. In proliferating myoblasts, the MyoD/CBF complex contains several chromatin modifying enzymes such as HDACs. In agreement with this, when overexpressed, CBFβ maintains histone H3 hypoacetylated, hypomethylated on lysine 4 and hypermethylated on lysine 9, on MyoD target promoters, along with HDAC1 recruitment, even in differentiation conditions. Finally, Runx1 is recruited to MyoD target genes preferentially in proliferating myoblasts, when these genes are repressed. Altogether, our data suggest that CBF transcription factor plays a pivotal role as a negative regulator of skeletal muscle terminal differentiation.

## Results

### CBF Subunits, Runx1 and CBFβ, Interact with MyoD in Proliferating Myoblasts

**Figure 1:**
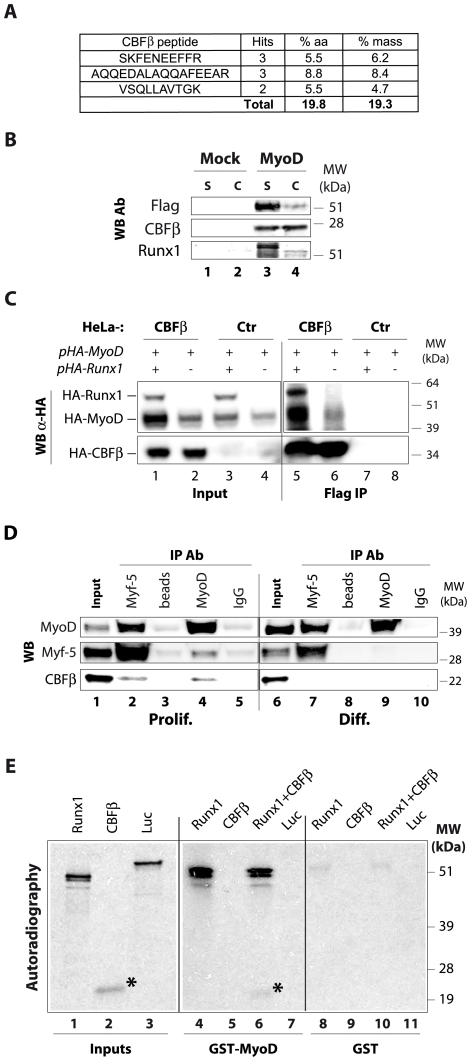
Runx1 and CBFβ interact with MyoD *in vitro* and in proliferating myoblasts. **A.** Peptide sequences identified by mass spectrometry in the MyoD complex corresponding to CBFβ protein. **B.** Western blot analysis of double-purified Flag-HA-MyoD (MyoD), or eluate from HeLa control cells (Mock) with the indicated antibodies (WB Ab). S: soluble; C: chromatin associated. **C.** HeLa cells stably expressing Flag-HA-CBFβ (eCBFβ) or HeLa control cells stably transfected with the empty vector (Ctr) were transiently transfected with expressing vectors for HA-tagged MyoD and/or HA-tagged Runx1. 24 hours post-transfection, cells were harvested and lysates were used for immunoprecipitation (IP) using Flag resin to precipitate Flag-HA-CBFβ. Precipitates were then subjected to western blot using HA Ab (WB α-HA) to simultaneously detect HA-Runx1, HA-MyoD, and Flag-HA-CBFβ (discriminated on the gel by their molecular weight). However, we have checked the identity of each HA-revealed band by using antibodies recognizing the native proteins (Figure S8). **D.** Nuclear extracts from proliferating (prolif.) or differentiating myoblasts (48 h, indicated as Diff.) were used for immunoprecipitation (IP) with antibodies (Ab) raised against MyoD (lanes 4 and 9) and Myf5 (lanes 2 and 7), with control beads (lanes 3 and 8) or with normal rabbit IgG (lanes 5 and 10) as negative controls. The resulting precipitates were then subjected to western blot analysis (WB) for the presence of MyoD, Myf5 and CBFβ. Input extracts were loaded to show endogenous protein levels (lanes 1 and 6). **E.** Runx1, CBFβ, or luciferase (Luc) were *in vitro* translated in the presence of ^35^S-Methionine (inputs on lanes 1-3, respectively) and incubated with equivalent amounts of GST-MyoD beads (lanes 4–7) or GST beads (lanes 8–11). GST pull-down was then conducted as described in the Material and Methods section, and the radiolabeled proteins were detected by autoradiography. *: CBFβ.

In an attempt to characterize MyoD protein partners, we carried out double-affinity purification of HA-Flag MyoD stably expressed in HeLa cells (see purification scheme on Figure S1). MyoD protein complex composition was then analyzed by mass spectrometry (MS) and western blot (WB). MS analysis of the purified protein complex revealed some already known partners of MyoD (Figure S2), such as Id, Pbx1, PC4 and E12/E47, and partners that had never been described to interact with MyoD. Indeed, MS analysis unveiled CBFβ protein within MyoD complex with a high number of peptides, covering almost 20% of its protein mass and amino acids content (Figure 1A). WB analyses confirmed that result and showed that Runx1 also co-purified with MyoD (Figure 1B).

We then performed a complementary experiment by transfecting HA-tagged MyoD and/or HA-tagged Runx1 into HeLa cells stably expressing Flag-HA-CBFβ (ectopic, eCBFβ). We showed that, indeed, MyoD co-precipitated with eCBFβ (Figure 1C, lane 6). Moreover, the simultaneous co-transfection of HA-tagged Runx1 resulted in its co-precipitation with CBFβ (Figure 1C, lane 5) and, more importantly, increased the MyoD co-precipitation (Figure 1C, compare lanes 5 and 6).

To further investigate the CBF/MyoD interaction, we turned to myogenic cells: the murine myoblastic cell line C2C12. Both CBFβ and Runx1 are expressed in the C2C12 myoblasts and their protein level do not vary significantly during differentiation (Figure S3). We found that MyoD, and the other myogenic determination factor Myf5, co-precipitated with CBFβ preferentially in proliferating compared to differentiating C2C12 myoblasts (Figure 1D).

To assay whether MyoD has the ability to interact directly with CBF, we performed GST pull-down experiments, which showed that GST-MyoD strongly interacts with Runx1 (Figure 1E, lane 4), but not with CBFβ (Figure 1E, lane 5). The interaction of MyoD with Runx1 was specific; we did not detect any Runx1 signal in the presence of GST protein alone (Figure 1E, lane 8) nor any luciferase signal with GST-MyoD (Figure 1E, lane 7). Interestingly, GST-MyoD interacts with CBFβ only in the presence of Runx1 (Figure 1E, compare lanes 5 and 6), in agreement with our previous findings (Figure 1C). These results show that MyoD interacts directly with heterodimeric transcription factor CBF, *via* the Runx1 subunit.

### The bHLH and the Transactivating Domains of MyoD, and the Transcription Regulation Domain of Runx1 Are Required for Their Interaction

**Figure 2:**
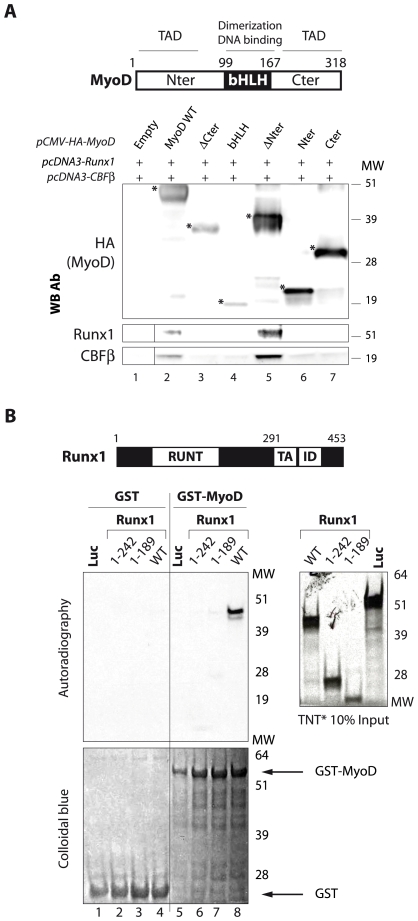
The domains of MyoD and Runx1 involved in their interaction. **A.** The bHLH domain and the C-terminal transactivating domain of MyoD are involved in the interaction with Runx1 and CBFβ. *Top panel*, Schematic diagram of MyoD functional domains, TAD: transactivation domain, bHLH: basic Helix Loop Helix. *Lower panel*, Runx1 and CBFβ interact with MyoD wild-type and with MyoD deletion mutant containing the bHLH and the C-terminal domain. *Lower panel*, expression vectors for wild-type MyoD and its deletion mutants were transfected in HEK 293 cells using Calcium phosphate precipitation as described in Material and Methods section. 48 h post-transfection, cells were lysed and lysates were subjected to anti-HA immunoprecipitation. Precipitates were then analyzed by western blotting using with the indicated antibodies (WB Ab). *: *specific bands*. **B.** The transcription regulation domain located in C-terminal part of Runx1 is required for the interaction with MyoD. *Top panel*, Schematic diagram of Runx1 protein. The Runt, transactivation (TA), and transcription inhibition (ID) domains are indicated. *Lower panel*, Runx1 and its deletion mutants, or luciferase (Luc) were *in vitro* translated in the presence of ^35^S-Methionine (inputs shown on the right panel) and incubated with equivalent amounts of GST-MyoD or GST agarose beads (normalization is shown in the lower panel). GST pull-down was then conducted as described in the Material and Methods section, and the radiolabeled proteins were detected by autoradiography.

In an attempt to delimit the domain of MyoD responsible for interaction with Runx1, we used HA-tagged mutants of MyoD transfected into HEK 293 cells. Anti-HA immunoprecipitation revealed an interaction of Runx1 and CBFβ with wild-type MyoD as expected, and only with a MyoD 82-318 mutant, which retains the bHLH and the C-terminal transactivating domains (Figure 2A). Runx1 and CBFβ failed to interact with truncated MyoD versions lacking either the bHLH, i.e., mutants Nter and Cter, or the C-terminal domain, i.e., mutants ΔCter, Nter and bHLH (Figure 2A). These experiments clearly show that the bHLH and the C-terminal transactivating domains are required for interaction with Runx1. Notably, MyoD deletion mutants that do not interact with Runx1 do not interact with CBFβ (Figure 2A). This result, combined with the results presented on Figure 1E, indicate that Runx1 is most likely the subunit that directly interact with MyoD.

To delimit the Runx1 domain involved in the interaction with MyoD, we performed a GST-pull down experiment (Figure 2B). Our results show that the transcription regulation domain located in the C-terminal part of Runx1 is required for the interaction with MyoD (Figure 2B). The interaction of MyoD with Runx1 was specific; we did not detect any Runx1 signal in the presence of GST protein alone nor any luciferase signal with GST-MyoD (Figure 2B).

### CBF Negatively Regulates Cell Cycle Exit and Terminal Differentiation in Skeletal Myoblasts

**Figure 3:**
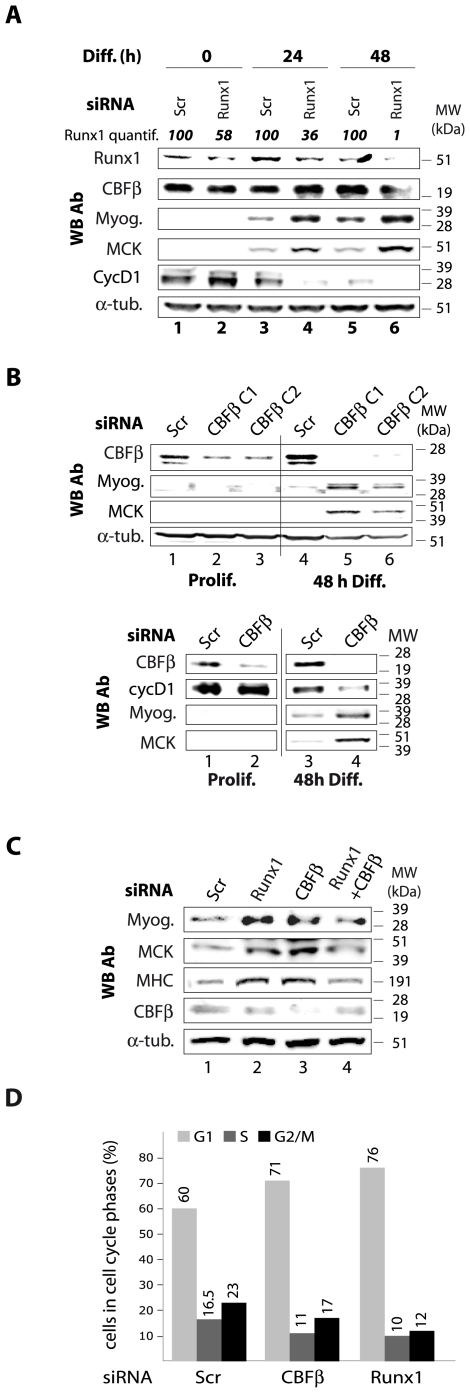
Downregulation of CBF subunits expression accelerates cell cycle exit and muscle terminal differentiation entry. **A.** C2C12 myoblasts were transfected with scrambled (Scr) or anti-Runx1 siRNAs. 48 h post-transfection (0 h, lanes 1–2), cells were placed in differentiation medium for 24 h (lanes 3–4) or 48 h (lanes 5–6). Cells were then analyzed by western blot with the indicated antibodies (WB Ab). The differentiation times are indicated in hours (h). Runx1 downregulation has been quantified (indicated as “Runx1 quantif.”) using Bio1D application (Vilber Lourmat). α-tubulin is used as a loading control. **B.** As in A, except that we used two different CBFβ siRNAs (C1, C2). **C.** As in A and B, except that we used proliferating primary myoblasts instead of C2C12 myoblasts, and combined Runx1 and CBFβ siRNAs (lane 4). Note that all the kinetic studies of differentiation were carried out in the same 10 cm diameter cell culture dish for each sample. **D.** FACS analysis of the cell cycle distribution of C2C12 myoblasts transfected with the indicated siRNAs. Cells were analyzed 48 h post-transfection. Scr: scrambled siRNA.

We used siRNAs to decrease Runx1 level in order to investigate its role in differentiating myoblasts (Figure 3A, *see Runx1 quantification*). Downregulation of Runx1 resulted in a more efficient differentiation (Figure 3A); both the expression of muscle markers and the proportion of multinucleated cells were higher in Runx1-depleted cells (Figure 3A and Figure S4A). In particular, myogenin and MCK (Muscle Creatine Kinase) were expressed at higher levels in Runx1-depleted cells (Figure 3A). Interestingly, cyclin D1 level decreased more rapidly when Runx1 is downregulated (Figure 3A). Similarly, CBFβ downregulation induced an accelerated differentiation (Figure 3B, top panel) and a more rapid decrease in cyclin D1 (Figure 3B, lower panel). Indeed, we could detect MCK expression in CBFβ-depleted myoblasts as soon as 24 h after the induction of differentiation (Figure 3B, top panel). These cells moreover exhibited larger myotubes (Figure S4B). We confirmed these results in primary myoblasts (Figure 3C).

In agreement with our previous results, we have found that Runx1 or CBFβ downregulation led to a significant decrease of S-phase cells proportion concomitant with an increase in G1-phase cells (Figure 3D). This suggests that CBF positively regulates myoblasts proliferation.

As for the specificity of the siRNAs, we obtained the same phenotype with three different siRNAs that target CBF: the two targeting CBFβ subunit and the one targeting Runx1 subunit isoforms. Thus, the observed effects are unlikely due to any off-target effect.

**Figure 4:**
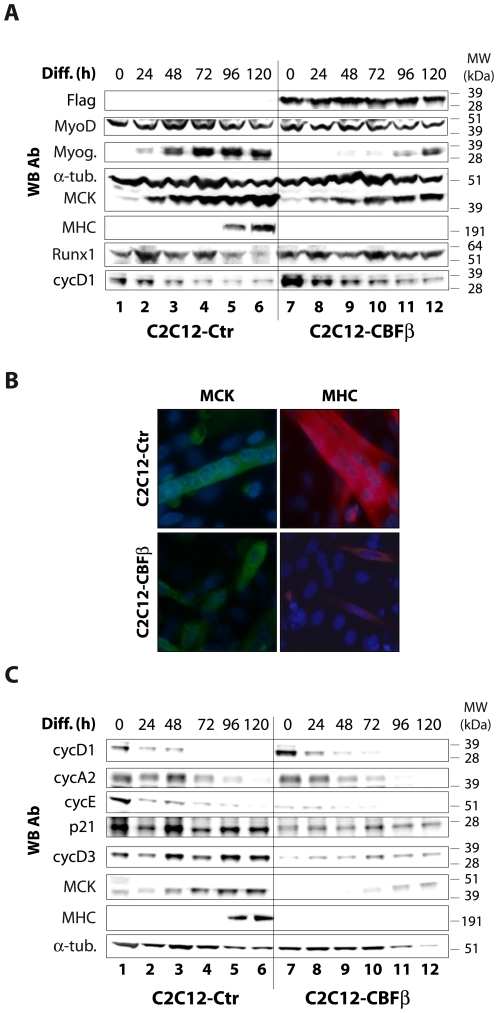
Overexpression of CBFβ delays cell cycle exit and muscle terminal differentiation. **A–C.** C2C12 cells stably overexpressing Flag-HA-CBFβ (C2C12-CBFβ) or control cells (C2C12-Ctr) were differentiated at the indicated times (in A and C, in hours), and analyzed by western blotting with the indicated antibodies (WB Ab) (A, C) or by immunofluorescence (IF) (63× magnification) (B). The kinetic studies were carried out in the same 10 cm diameter cell culture dish (A, C). IF experiments using anti-MCK or anti-MHC antibodies were performed 48 h and 72 h respectively after induction of differentiation. Cells were DAPI-stained prior to fluorescent microscopy analysis (63× magnification).

To complete our analysis, we studied the effect of CBFβ overexpression on terminal differentiation. Overexpression of CBFβ in C2C12 myoblasts (C2C12-CBFβ) was well tolerated and did not lead to morphological abnormality (Figure S5). However, in differentiation conditions, C2C12-CBFβ cells showed a delay in cell cycle exit, as measured by early-G1 phase cyclin D1 level that decreased with a 24 to 48 h delay compared to control cells (Figure 4A), but not that of late-G1 cyclins A and E (Figure 4C). Note that proliferating C2C12-CBFβ cells contain more cyclin D1 protein then the control cells (Figure 4A), but not cyclins A2 and E (Figure 4C). The delayed cell cycle exit correlated with delayed expression of muscle markers such as myogenin (48 h delay), MCK (24 h delay), and MHC (Myosin Heavy Chain, not detected at 120 h) (Figure 4A). In contrast to control cells, C2C12-CBFβ cells exhibited smaller and mainly mononucleated myotubes (Figure 4B and Figure S5) with low expression of MCK and MHC (Figure 4B) in differentiation conditions. This suggests that differentiation kinetics was not completely impaired but greatly delayed when CBFβ was overexpressed. Further analysis of cell cycle regulators expression showed that, in addition to a delayed decrease in cyclin D1 (Figure 4A and C), cyclin D3 and p21 expression is delayed in C2C12-CBFβ cells compared to C2C12 control cells (Figure 4C).

Altogether, these results suggest that CBF plays a dual role during skeletal muscle differentiation by regulating cell cycle withdrawal and expression of muscle markers.

### CBF Transcription Factor Is Located to MyoD Early Target Genes and Regulates Negatively Their Expression

**Figure 5:**
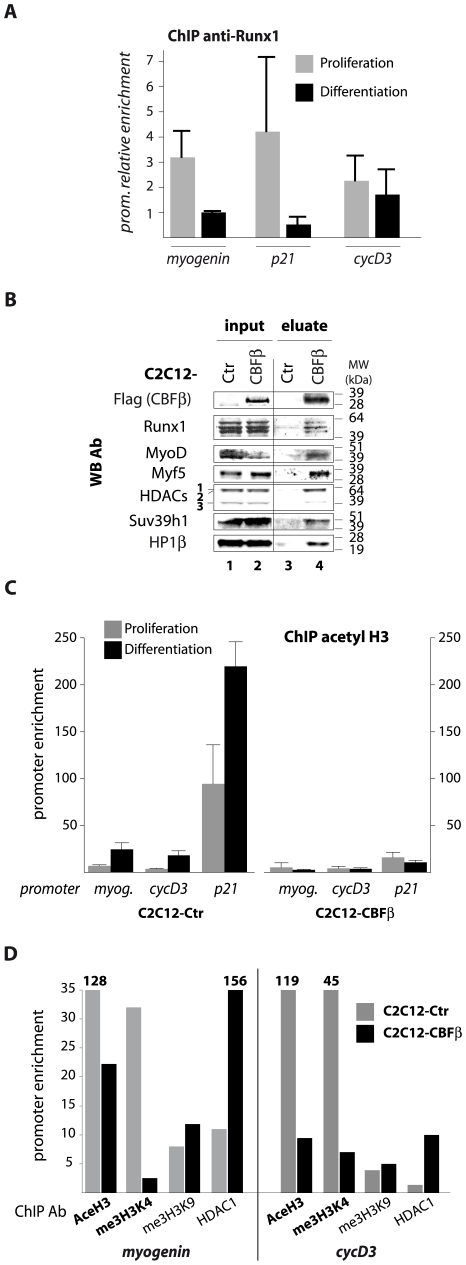
CBF acts at the chromatin level to regulate MyoD target genes. **A.** ChIP using anti-Runx1 Ab were performed from either proliferating (grey bars) or differentiating myoblasts (black bars). We quantified copy numbers of the *myogenin*, *p21* and *cyclin D3* promoter regions harboring the MyoD and Runx1 target sequences, compared to *36B4* gene, which was used as a reference gene. Results are the mean of 3 independent experiments. **B.** Western blot analysis with the indicated antibodies (WB Ab) of Flag-purified Flag-HA-CBFβ stably expressed in C2C12 cells (CBFβ), or from C2C12 control cells (Ctr). Both inputs are probed in lanes 1–2 and eluates are shown in lanes 3–4. **C.** Chromatin immunoprecipitation (ChIP) experiments using anti-acetyl H3 antibody were performed from either proliferating (grey bars) or differentiating (black bars) C2C12 control (left) and C2C12-CBFβ (right) cells. We quantified copy numbers of the *myogenin* (*myog*.), *cyclin D3* and *p21* promoter regions harboring the MyoD target sequences. Results are the mean of three measurements. **D.** ChIP experiments using antibodies against acetyl histone H3 (AceH3), trimethylated histone 3 lysine 4 (me3H3K4), trimethylated histone 3 lysine 9 (me3H3K9), Histone Deacetylase 1 (HDAC1) were performed from differentiating C2C12 control (grey bars) or C2C12-CBFβ (black bars) cells (48 h differentiation time). We quantified copy numbers of the *myogenin* and *cyclin D3* promoter regions harboring the MyoD target sequences. Results with the transcription activating marks (AceH3 and me3H3K4) were normalized using the expressed housekeeping gene *36B4*, while the repressive marks (me3H3K9 and HDAC1) were normalized using the repressed major satellite repeats. Results are the mean of three measurements.

The fact that CBF is a transcription factor which interacts with MyoD preferentially in proliferation conditions, led us to investigate whether it would be targeted to MyoD target genes to repress their transcription. Using an *in silico* approach, we first found that on early target gene promoters of MyoD, Runx1 and MyoD binding sites were adjacent (Figure S6A). In order to test the effective recruitment of Runx1 onto MyoD target promoters, we performed ChIP experiments. Our results showed a preferential enrichment in Runx1 on *myogenin*, *p21* and *cycD3* promoters in proliferating compared to differentiating myoblasts (Figure 5A). These MyoD target genes are expressed early in differentiating but not in proliferating myoblasts (Figure S6B).

Our ChIP assays showed that Runx1 was not located on late target genes of MyoD, such as *Desmin*, *MHC* and *MCK* (data not shown), while it was on early muscle differentiation genes *myogenin*, *p21* and *cycD3*. In addition, we did not find Runx1 binding sites adjacent to E-boxes on late MyoD target genes' promoters. These findings suggest that Runx1 would essentially regulate early events of skeletal muscle terminal differentiation. Taken together, our results strongly suggest that Runx1 could be recruited onto MyoD early target genes to regulate negatively their expression in proliferating myoblasts.

To gain insights into the mechanism of action of CBF on MyoD target genes, we purified CBFβ protein complex from proliferating C2C12-CBFβ cells *via* its Flag tag. As expected, CBFβ co-purified with the myogenic factor MyoD and with its dimerization partner Runx1 (Figure 5B). The other partners that co-purified specifically with CBFβ are proteins known to be involved in transcriptional repression: the histone 3 lysine 9 (H3K9) methyltransferase Suv39h1, the heterochromatin protein HP1β, and the histone deacetylases HDACs 1, 2 and 3 (Figure 5B). These interactions could be mediated by Runx1. Indeed, these proteins are already known partners of Runx1 on the one hand [30], and repressors of MyoD activity on the other hand [31]–[33].

Given the association of CBFβ with chromatin-modifying enzymes, we studied the chromatin status of three target gene promoters of MyoD in differentiating C2C12-CBFβ using chromatin immunoprecipitation (ChIP). Our results showed that, in contrast to control cells in which histone H3 acetylation (a mark associated with transcription activation) on *myogenin*, *cyclin D3* and *p21* promoters increased in differentiation compared with proliferation conditions, histone H3 acetylation levels at these promoters did not vary in C2C12-CBFβ cells (Figure 5C). More generally, in differentiation conditions, we found that activating marks (histone H3 acetylation, histone H3 lysine 4 tri-methylation) are abnormally lower on *myogenin* and *cyclin D3* promoters in C2C12-CBFβ cells compared to control cells (Figure 5D). Concomitantly, repressive marks (histone H3 lysine 9 tri-methylation, presence of HDAC1) are higher (Figure 5D).

These results are in agreement with our findings showing that CBF associates with chromatin modifying enzymes, such as HDACs, Suv39h1 and HP1β (Figure 5B), which are known to repress MyoD activity in proliferating myoblasts. Thus, CBF has an effect on the chromatin structure of three early target genes of MyoD and contributes to maintain a repressive chromatin state.

### Runx1 Represses MyoD Transcriptional Activity

**Figure 6:**
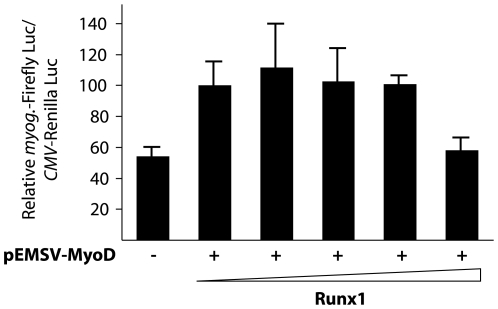
Runx1 represses MyoD-mediated transcription. Co-transfection into HeLa cells with a *myogenin* promoter-driven Firefly luciferase reporter plasmid (kind gift of V. Sartorelli, NIH) without or with a fixed amount of MyoD expression vector (800 ng), and increasing amounts of vector expressing Runx1. The quantities of Runx1 expression vector used were: 0, 30, 60, 150, and 300 ng. The total amount of plasmid was normalized when necessary to 300 ng with the empty vector. The inhibitory effect of Runx1 was specific, indeed, it was not seen with a Renilla luciferase expression under a *CMV* promoter (used as a normalization control for the transfection). Results are the mean of 2 independent experiments performed each in triplicates.

To investigate the effects of Runx1 on MyoD transcriptional activity, we used a luciferase reporter gene under the control of the *myogenin* promoter, which is a direct target promoter of MyoD that harbors a Runx-binding site adjacent to MyoD-binding site (Figure S6A). Co-transfection experiments were performed in HeLa cells line that does not express MyoD endogenously. We observed that the co-transfection of Runx1-expressing plasmid together with MyoD expression vector resulted in the inhibition of *myogenin* promoter activity in a dose-dependent manner (Figure 6). The inhibitory effect of Runx1 is specific and is MyoD-dependent. Indeed, it was not seen with Renilla–luciferase expression under a *CMV* promoter (used as a normalization control for transfection efficiency). Thus, Runx1 inhibits MyoD activity.

## Discussion

Here we show that CBF transcription factor, composed of Runx1 and CBFβ subunits, is expressed in proliferating myoblasts where it interacts with MyoD. Modulating the expression levels of either Runx1 or CBFβ impaired cell cycle exit and terminal myogenic differentiation.

### Runx1/CBFb Interact with MyoD

Mass spectrometry analysis revealed that CBFβ is part of MyoD complex. Further western blot analyses of the same MyoD complex revealed the presence of Runx1 subunit. We have narrowed our study to Runx1 because of all the three Runx proteins, to our knowledge, Runx1 is the only one linked to skeletal muscle [20]. In addition, *Runx1* is one of the MyoD target genes in myoblasts [34]. Among the other Runx members, Runx2 is mainly involved in osteogenesis, and it has already been shown that Runx2 expression is not detectable in myoblasts [35]. Moreover, ectopic expression of Runx2 in myoblasts triggers osteogenic transdifferentiation [35]–[37]. Finally, concerning Runx3, it has been shown that it is not expressed in skeletal muscle and is mainly linked to neurogenesis [38]–[42]. Thus, we explored the role of Runx1/CBFβ during muscle skeletal terminal differentiation. However, Runx1 has been reported to play an important role in protecting denervated, fully differentiated, myofibers from atrophy and autophagy [20]. In this paper [20], Runx1 has been assigned a role of a growth-promoting factor of muscle cells to limit muscle wasting. In this very elegant study, the authors generated *knock-in* homozygous mice carrying inactive *Runx1* in skeletal muscle cells. However, *Runx1* inactivation was conducted under the indirect control of *MCK* promoter, which is active only at late stages of terminal differentiation. Thus, with this system, we cannot see the effect of *Runx1* inactivation on early stages of terminal differentiation, where *MCK* promoter is not yet active.

### Runx1 and CBFb Regulate Cell Cycle Exit and Terminal Differentiation

Runx1 or CBFβ downregulation in myoblasts induced an accelerated cell cycle exit. Indeed, cyclin D1 protein disappears 24 h earlier in differentiation conditions. In addition, Runx1 or CBFβ downregulation led to a an increase in G1-phase cells, suggesting that CBF promotes myoblasts proliferation. Conversely, CBFβ overexpression delayed the early-G1 phase cyclin D1 disappearance in differentiation conditions (cyclin D1 is still detectable 72 hours after the induction of differentiation), but not that of late-G1 and early-S phase cyclins A2 and E1. Note that proliferating C2C12-CBFβ cells contain more cyclin D1 protein, but not cyclins A2 and E, than the control cells. Together, these results suggest that CBFβ overexpression impacts on early G1 markers. In addition, expression of the cell cycle exit regulators p21 and cyclin D3 [43], [44] is delayed in differentiating CBFβ-overexpressing cells, suggesting a delayed or impaired cell cycle exit and terminal differentiation entry. In agreement with this, our ChIP results showed that in proliferating myoblasts, CBF (*via* Runx1) is recruited to repressed *p21* and *cyclin D3*, which encode cell cycle exit regulators [43], [44]. Together, these results suggest that CBF regulates positively proliferation, and negatively terminal differentiation, of skeletal myoblasts. Thus, CBF impacts on the proliferation/differentiation switch in myoblasts (see model on Figure S7).

In differentiation conditions, C2C12-CBFβ cells showed delayed molecular differentiation (expression of muscle markers), and delayed appearance of myotubes that are abnormally small and mainly mono-nucleated. One alternative explanation is that these cells managed to differentiate correctly, although in a delayed manner, but exhibited a specific block in fusion and multi-nucleation. Indeed, CBFβ subunit is retained in the cytoplasm by cortical filamins [45]. In muscle cells notably, structural proteins and cell adhesion proteins are required for the reorganization of the cell cytoskeleton during cell fusion to form myotubes [46]. Some studies do suspect a cytoplasmic role for CBFβ [47]. Thus, CBFβ overexpression in myoblasts, especially in the cytoplasm, could have a role in impairing the correct cytoskeleton reorganization during fusion. This could explain the phenomenon we observe in differentiating CBFβ-overexpressing myoblasts. Moreover, we observed a very low expression of myogenin transcription factor in myoblasts overexpressing CBFβ and since that myogenin is involved in cell fusion, this is an alternative explanation of the observed the mono- or di-nucleated myotubes.

### CBF Regulates Muscle Differentiation via a Direct Interaction between MyoD and the Runx1 Subunit

Our results revealed that the role of CBF in myoblasts is likely to be partly mediated through direct interaction with MyoD. GST pull-down experiments showed that MyoD interacts directly with Runx1 subunit, but not with CBFβ. This is confirmed by the use of MyoD deletion mutants in living cells. Indeed, MyoD deletion mutants that fail to interact with Runx1 do not interact neither with CBFβ. MyoD/Runx1 interaction implicates the bHLH and the C-terminal transactivating domains of MyoD, and the transcription regulation domain of Runx1.

The preferential association of MyoD and CBF in proliferating myoblasts could mean that CBF might be acting as a negative co-factor of MyoD. Indeed, we provide evidence that CBF is recruited to early MyoD target genes, *via* Runx1, in proliferating myoblasts, where MyoD is mainly associated with transcriptional repressors [24], [26], [31]. This suggests that CBF may serve for assembly of a transcription repression complex at early MyoD target genes such as *myogenin*, *p21* and *cyclin D3* (see our model on Figure S7). As for example, such a mechanism could be involved in the repression of the skeletal muscle *acetylcholine receptor* gene, which contains a repressive E-box that mediates its repression in proliferating myoblasts [48]. In agreement with this, we found that in proliferating myoblasts, CBF associates with many chromatin modifying enzymes, such as Histone Deacetylases (HDACs 1, 2 and 3), the histone H3 lysine 9 (H3K9) methylase Suv39h1, and Heterochromatin Protein beta (HP1β), which are known to repress MyoD activity in proliferating myoblasts [24], [31], [49], [50]; and already known to interact with Runx1 [30], [51], [52]. In agreement with this, in myoblasts overexpressing CBFβ, histone H3 acetylation and its trimethylation on lysine 4 (marks of active transcription) are delayed on early MyoD target genes in differentiation conditions. Concomitantly, these genes remain abnormally marked by histone repressive marks (me3H3K9, HDAC1).

Interestingly, although MyoD is expressed both in proliferating and differentiating cells, we found that the interaction between MyoD and CBF was lost in differentiating cells. In addition, we showed that overexpression of CBFβ in myoblasts led to stabilization of Runx1 subunit that could more efficiently repress MyoD transactivating activity, which induces a delay in terminal differentiation. In agreement with this, we have shown that Runx1 represses MyoD activity in a gene reporter assay.

We did not succeed to show the concomitant presence of Runx1 and MyoD on MyoD target genes, given that it has previously been demonstrated that in proliferating conditions, only a small fraction of MyoD contributes to the repressive remodeling of its target genes, prior differentiation. Alternatively, Runx1 could prevent the proper binding of MyoD and the recruitment of the transcriptional machinery. Notably, the displacement of Runx1 in differentiating conditions is concomitant with a strong binding of MyoD to its target promoters (data not shown).

MyoD and Runx1 are both subject to post-translational modifications. Notably, MyoD is phosphorylated during the cell cycle while it becomes acetylated during differentiation. Runx1 can also be phosphorylated, acetylated or methylated, while these modifications still need to be characterized in muscle cells and during muscle differentiation. We propose that these modifications could favor or impair MyoD interaction with Runx1, respectively.

### Conclusion

Our findings concerning the role of CBF in the regulation of the proliferation/differentiation balance are in agreement with several reports. CBF was indeed implicated in skin epidermis and hair follicle differentiation [11], [12], as well as in neuronal differentiation [8], [9]. These data support an emerging role for Runx proteins in cell fate regulation in many cell lineages. Furthermore, MyoD was also shown to regulate osteogenic differentiation [53]. In addition, it has been shown that muscle satellite cells can differentiate into osteocytes or adipocytes under some conditions [54], [55]. Thus, our results point to a model in which CBF and myogenic bHLH protein families could act in concert to induce cell-lineage-specific gene expression, dependent on the extra-cellular stimuli.

In summary, we propose that CBF transcription factor might participate in recruiting chromatin modifying enzymes to repress MyoD early target genes by locally inducing a repressive chromatin structure. Our data reveal a new critical role of CBF in the regulation of the balance between proliferation and differentiation in skeletal muscle cells. They also demonstrate a new mechanism of repression of differentiation genes in proliferating myoblasts.

## Methods

### Cell Culture

C2C12, HEK 293 and HeLa-S3 cells were cultured under standard conditions. C2C12 cells and mouse primary myoblasts were cultured and differentiated as described in: [56].

### Stable Cell Lines Establishment and Plasmid Construction

A HeLa cell line stably expressing MyoD was established with a transgene encoding for full-length MyoD; and HeLa and C2C12 cell lines expressing CBFβ were established with a transgene encoding for full-length CBFβ. The transgenes were tagged with double-HA (Haemagglutinin) and double-FLAG epitopes at the N-terminus as described in [57].

Control cell lines transduced with the empty vector were also established. Murine CBFβ cDNA (a kind gift from Dr Nancy A. Speck) was amplified by PCR with specific primers with protruding restriction sites (fw-Pspx1: CCGCTCGAGCCGCGCGTCGTCCCGGG, rev-Not1: ATTCTATATGCGGCCGCTAACGAAGTTTGAGATCATCG, and sub-cloned into the XhoI-NotI sites in the pREV retroviral vector after Pspx1 and Not1 digestion (Pspx1 is compatible with Xho cloning site in the pRev vector) [57], [58].

### Protein Complex Purification

Flag-HA-MyoD complex purification from HeLa-MyoD cells was performed as described in: [31]. Briefly, we used retroviral transduction strategy to establish HeLa-S3 cell lines expressing double tagged Flag-HA-MyoD [31], [57], [58], or a control cell line transduced with the empty pREV vector has been established. We carried out double-affinity purification of Flag-HA-MyoD from HeLa cells (Figure S1), using either nuclear soluble or chromatin fractions. For this, cells were resuspended in a hypotonic buffer (10 mM Tris-HCl pH 7.65; 1.5 mM MgCl_2_; 10 mM KCl) and disrupted with 20 strokes of a tight-fitting Dounce homogenizer. The cytosolic fraction was separated from nuclei by 7 min centrifugation at 4°C at 9000 rpm. The nuclear soluble fraction was obtained by incubation of the nuclear pellet in a high salt buffer (900 mM NaCl, 20 mM Tris pH 7.65, 25% glycerol, 1.5 mM MgCl_2_, 0.2 mM EDTA), to get 300 mM NaCl, for 30 min at 4°C and centrifugation at 10,000 rpm. The resulting pellet, which corresponds to chromatin fraction, was resuspended and digested with micrococcal nuclease (Sigma, Saint-Quentin Fallavier, France), until it consisted primarily of mononucleosomes [57]. Nuclear soluble and chromatin fractions were then ultracentrifugated at 32000 rpm for 1 h at 4°C. Tagged-MyoD complex were then purified using anti-FLAG antibody immobilized on agarose beads (Sigma). After elution with the FLAG peptide (Ansynth, The Netherlands), the bound complexes containing nucleosomes were further affinity-purified on anti-HA antibody-conjugated agarose (Sigma) and eluted with the HA peptide (Ansynth, The Netherlands). Double-immunopurified complexes were resolved on 4–12% SDS-PAGE bis-Tris acrylamide gradient gel in MOPS buffer (Invitrogen), and stained using either the SilverQuest kit (Invitrogen, Cergy-Pontoise, France) [31], or with Colloidal blue (Invitrogen) for mass spectrometry (MS) analyses. In the latest, bands corresponding to proteins were cut from the gel, trypsin-digested using 0.4 mg of sequencing-grade trypsin (Promega, Charbonnières, France), and identified by MS analysis.

To purify CBFβ complex from C2C12 cells, 3 grams of C2C12-CBFβ cell pellet were used to purify tagged CBFβ using a simple-affinity purification method using Flag resin.

### Preparation of Nuclear Extracts

Cells were scraped in a minimal volume of PBS and centrifuged 2 min at 400 g. The pellet was resuspended in 5 volumes of: 20 mM HEPES pH 7, 0.15 mM EDTA, 0.15 mM EGTA, 10 mM KCl, then lysed by addition of NP-40 up to 4.5%. Nuclei were immediately neutralized with addition sucrose buffer (50 mM HEPES pH 7, 0.25 mM EDTA, 10 mM KCl, 70% (m/v) sucrose). After centrifugation (5 min, 2000 g), nuclei were suspended in glycerol buffer (10 mM HEPES pH 8, 0.1 mM EDTA, 100 mM NaCl, 25% glycerol) to remove any trace of cytosolic components and centrifuged again. The nuclei were then resuspended in sucrose buffer n°2 (20 mM Tris pH 7.65; 60 mM NaCl; 15 mM KCl; 0.34 M Sucrose) then lysed in a final concentration of 250 mM NaCl using High Salt Buffer (20 mM Tris pH 7.65; 0.2 mM EDTA; 25% glycerol; 900 mM NaCl; 1.5 mM MgCl_2_). The lysates were sonicated 3 times for 15 s with the BioRuptor (Diagenode, Liège, Belgium) on “High”, then centrifuged 10 min at 13000 rpm to harvest the total nuclear extracts (supernatants). Protein concentration for each sample was estimated with BCA kit (Perbio, Brebières, France).

### Transient Transfections, Flag-Affinity Precipitation of Flag-HA-CBFb, HA-MyoD Precipitation

For plasmid transfection, 25 µg of pRcCMV-HA-Runx1 (kind gift of Dr I. Kitabayashi, Japan), pCMV-HA-MyoD or pRC-CMV backbone were transfected into HeLa-CBFβ cells, using calcium phosphate pH 7.12, and Flag IPs was performed 24 h post-transfection (results presented on Figure 1C). Each IP was performed with 1.5 mg of total nuclear extracts and with 25 µL stock of ssDNA and BSA-pre-blocked Agarose Flag M2 resin from Sigma. IP was performed on wheel overnight at 4°C. Resin was then washed 5 times with TEGN buffer (20 mM Tris pH 7.65; 0.1 mM EDTA; 10% glycerol; 150 mM NaCl; 0.5% NP-40) and eluted by competition with high-purity Flag peptide at a final concentration of 0.2 mg/ml. The resin-free eluate was retrieved using Clean-up Post reaction columns (Sigma).

For interaction experiments using MyoD deletion mutants, HEK 293 cells were transiently transfected using calcium phosphate at pH 7.12. We used for a 10-cm dish 5 µg of a pCMV-3HA-MyoD or its deletion mutants (Cter 241-318, Nter 1-66, bHLH 82-172, ΔCter 1-240, ΔNter 82-318), or the empty vector, along with 5 µg of pcDNA3-Runx1 and 5 µg of pcDNA3-CBFβ vectors. 48 h post-transfection, cells were lysed in lysis buffer (300 mM NaCl, 50 mM Tris–HCl, pH 7.5, 0,4% NP-40, 10 mM MgCl_2_) to extract proteins. Anti-HA immunoprecipitation was then performed as described above.

### siRNA Transfection

siRNAs were purchased from Sigma (Saint-Quentin Fallavier, France) and were transfected using Hi-Perfect reagent (Qiagen, Courtaboeuf, France) according to the manufacturer recommendations. We usually transfect 0.2 µmol of siRNA per 100 mm cell culture dish. CBFβ targeting siRNA sequences used are: C1: CCGGGAAUAUGUCGACUUA, and C2: UAACUUAGGUGGCGGUGAU; Runx1 siRNA: CUGUGAAUGCUUCUGAUUU; and the scrambled siRNA: ACUUAACCGGCAUACCGGCTT.

### Immunoprecipitation of Endogenous Proteins

For IP, we usually use 2 µg of antibodies, 10 µL protein A/G Sepharose beads from Perbio and 1.2 mg of nuclear extracts from C2C12 cells, or 0.5 mg from primary myoblasts. Elution was performed with 40 µl of 0.1 M glycine pH 2.5, 15 min at 25°C, the eluate was recovered using Spin cleaning-up post-reaction column (Sigma). Acidity was neutralized with Tris pH 8.0 before adding loading buffer.

### Western Blotting

For western blotting, protein samples were resolved on pre-cast NuPage 4–12% bis-Tris acrylamide gradient SDS-PAGE gel (Invitrogen, Cergy-Pontoise, France). Proteins were then transferred onto nitrocellulose membrane during 1 h at 400 mA in transfer buffer (25 mM Tris, 150 mM Glycine, 0.1% SDS and 20% methanol). Membranes are blocked 1 hour in PBS-0.2% Tween, 10% skimmed milk and incubated overnight at 4°C with primary antibodies. Membranes were incubated with the appropriate secondary antibodies coupled to HRP and revealed using West Dura from Pierce (Perbio, Brebières, France) and ChemiSmart 5000 system (Vilber Lourmat, Marne-La-Vallée, France).

### Plasmids, GST Fusions and GST Pull-Down

GST and GST-MyoD plasmid constructs were expressed in *Escherichia coli* strain BL21 and purified using glutathione-sepharose beads according to the manufacturer (Sigma, Saint Quentin-Fallavier, France). Purified proteins were quantified by coomassie staining after SDS–PAGE separation. *In vitro* transcription and translation (TNT) of pcDNA3-Runx1 and its deletion mutants aa 1-189 and 1-242, pcDNA3-CBFβ and luciferase were performed with Riboprobe *in vitro* transcription systems (Promega, Charbonnières, France) in the presence of ^35^S-labelled methionine.

Agarose beads coated with equal amounts of GST or GST-MyoD (1 µg) were incubated with 10 µL of radioactive TNT reaction in reaction buffer (50 mM Tris pH 7.6, 150 mM NaCl, 0.1% Triton) during 2 h at 4°C. Beads were washed 5 times with wash buffer (50 mM Tris pH 7.6, 300 mM NaCl, 0.5% Triton 100), resuspended and proteins resolved by SDS-PAGE gel and revealed by autoradiography.

### Immunofluorescence

Cells were cultured in Labtecks permanox (Falcon) and fixed briefly with 4% formaldehyde in PBS. Residual formaldehyde was neutralized with 0.1 M Glycine pH 8.0, and washed with PBS. Cells were permeabilized and blocked using 1% BSA, 1% goat serum, 0.3% Triton-X100 in PBS. Primary and secondary antibodies were diluted in the permeabilizing/blocking solution and were washed with 0.3% Triton-X100 in PBS. Nuclei are stained with DAPI and the glass lid is fixed using an anti-fading polymerizing media from DakoCytomation (Dako, Trappes, France).

### Antibodies

The anti-MyoD (C-20), anti-myogenin (M-225), anti-Myf5 (C-20), anti-CBFβ (FL-182), anti-cyclin A2 (C19, sc-596), anti-cyclin D1 (72-13G, sc-450), anti-cyclin D3 (C-16, sc-182), anti-cyclin E (sc-25303), anti-p21 (C-19, sc-397) and normal rabbit IgG antibodies were all purchased from Santa Cruz (Santa Cruz, CA, USA). Rabbit polyclonal anti-Suv39h1 (07-550), anti-trimethyl histone 3 lysine 9 (07-442) and rabbit anti-acetyl histone H3 (06-599) antibodies were obtained from Upstate Biotech (Lake Placid, NY, USA). Anti-HP1β (1MOD1A9AS) was from Euromedex (Souffelweyersheim, France). Anti-HDAC1 (pAB-053-050) was from Diagenode (Liège, Belgium). Anti-trimethyl H3K4 was from Abcam (Paris, France). Rabbit polyclonal anti-MCK antibody was developed by Dr H. Ito [59]. Anti-Flag and anti-α-tubulin antibodies were purchased from Sigma (Saint-Quentin Fallavier, France). Rat anti-HA antibody was purchased from Roche (Meylan, France). Mouse anti-Runx1 antibody (MAB10062) was purchased from Millipore (Saint Quentin en Yvelines, France) and mouse anti-HDAC 1-3 antibody (611125) from BD Biosciences (Le Pont de Claix, France). Goat anti-rat IgG Alexa-488-conjugated, anti-rabbit IgG Alexa-488 were from Invitrogen (Cergy-Pontoise, France) and anti-mouse IgG TRITC (T7657) were from Sigma (Saint-Quentin Fallavier, France).

### FACS Analysis

C2C12 were transfected with the siRNAs as indicated in the Material and Method section. 48 hours post-transfection, cells were washed with PBS, then scraped in 500 µL of PBS. Cells were kept on ice while 4,5 ml of ethanol 70% were added. Then cells are kept at least overnight at −20°C. Propidium iodide (PI) staining proceeds as follows: cells are centrifuged and the pellet is washed with PBS. Cells are then centrifuged and resuspended in 2 mL PI solution (PI 25 ng/ml, RNase 200 ng/ml, Triton 0,1%) 30 minutes and kept in the dark. Cells are homogeneized by vortexing before analysis. We worked on a Beckman and Coulter FACS apparatus and we counted at least 3000 events for each condition.

### Chromatin Immunoprecipitation (ChIP)

ChIP protocol and primers have been described in: [56]. The yet unpublished primers used are: Myogenin fw: GAATCACATGTAATCCACGGA, rev: ACGCCAACTGCTGGGTGCCA. Cyclin D3 fw: CTGCTTGCCTCTGTCTTCA; rev: GACCCATGTCAGATGACTC. 36B4 fw: ATGTGCAGCTGATAAAGACTGG; rev: CTGTGATGTCGAGCACTTCAG.

### Gene Reporter Assays

HeLa cells at 60% confluence were co-transfected by Calcium Phosphate co-precipitation with a *myogenin* promoter-driven Firefly luciferase reporter plasmid (kind gift of V. Sartorelli, NIH) without or with a fixed amount of MyoD expression vector (800 ng), and increasing amounts of vector expressing Runx1. The quantities of Runx1 expression vector used were: 0, 30, 60, 150, and 300 ng. The total amount of plasmid was normalized when necessary to 300 ng with the empty vector. A Renilla luciferase expression under a *CMV* (cytomegalovirus) promoter was used as a normalization control for the transfection. 24 h post-transfection, cells were lysed in a reporter lysis buffer (Promega, Charbonnières, France). Luciferase activity was determined using Dual-Luciferase Reporter Assay System (Promega). Firefly luciferase activity was then normalized to the level of Renilla luciferase and to the total protein amount.

## Supporting Information

Figure S1 Schematic representation of the purification protocol used to purify the MyoD complex from HeLa cells.
(0.38 MB EPS)

Figure S2 MyoD known partners identified by mass spectrometry in the MyoD complex.
(0.05 MB DOC)

Figure S3 Expression of Runx1 and CBFβ proteins during muscle terminal differentiation. Cellular extracts from proliferating or differentiating C2C12 myoblasts (left panel) or from mouse primary myoblasts (right panel) were subjected to western blot analyses for the expression of CBFβ, Runx1, MyoD, myogenin (Myog.) and Muscle Creatine Kinase (MCK). α-tubulin is detected as a loading control. Differentiation times are indicated in hours on the top of each panel. Lanes 1 and 7 correspond to proliferating cells.
(0.57 MB EPS)

Figure S4 Downregulation of CBF subunits expression accelerates muscle terminal differentiation entry. A. C2C12 myoblasts were transfected with control siRNA (Scrambled, Scr) or with Runx1 siRNA as indicated. 48 hours post-transfection (Proliferation) cells were placed in differentiation medium (Differentiation) for 86 hours. Cells were then analyzed by microscopy (10× magnification). B. As in A, except that we used CBFβ siRNA.
(11.55 MB EPS)

Figure S5 C2C12-CBFβ cells characterization. A. Expression level of CBFβ in C2C12 control (ctr) and in C2C12-CBFβ, in proliferating myoblasts (0 h) or at the indicate differentiation times (in hours), as measured by western blot using anti-CBFβ antibody. ex.: exogenous; end: endogenous. B. C2C12 cells stably overexpressing Flag-HA-CBFβ (C2C12-CBFβ) or control cells (C2C12-Ctr) were differentiated for 72 hours and analyzed by light microscopy (10× magnification).
(10.67 MB EPS)

Figure S6 A. Runx1 and MyoD binding sites found in silico on MyoD target genes myogenin and p21. B. Western blot analysis (with the indicated antibodies) of cell extracts used for the ChIP experiment presented on Fig. 5A. α-tubulin (α-tub.) is used a loading control.
(0.52 MB EPS)

Figure S7 Proposed model of skeletal muscle terminal differentiation regulation by CBF and MyoD. Note that in muscle system, proliferation inhibition or cell cycle exit is a pre-requisite to terminal differentiation. Thus, cell cycle exit regulators, such as p21 and cycD3, are activated early during terminal differentiation. Muscle specific early markers, such as myogenin, are also activated before late muscle markers. In proliferating myoblasts, Runx1/CBFβ (CBF dimer) proteins repress MyoD target genes, possibly via a direct interaction with MyoD. Thus, in proliferating myoblasts, CBF binds early MyoD target genes via Runx1 subunit and recruits chromatin modifying enzymes such as HDAC1, Suv39h1 and HP1. Upon triggering of terminal differentiation, CBF dissociates from the promoters and MyoD recruits activating chromatin modifying enzymes, such as HATs. HDAC1: Histone Deacetylase 1; HAT: Histone acetyltransferase.
(0.54 MB EPS)

Figure S8 Control of the anti-HA western blot results presented on Figure 1C using antibodies against native proteins: MyoD, CBFβ and Runx1.
(0.53 MB EPS)
