# Supplementary material for: The Core Binding Factor CBF Negatively Regulates Skeletal Muscle Terminal Differentiation
Source: PLoS One. 2010 Feb 25;5(2):e9425. doi: 10.1371/journal.pone.0009425 (PMC2828485; doi:10.1371/journal.pone.0009425)
Supplement: Figure S2 — MyoD known partners identified by mass spectrometry in the MyoD complex. (0.05 MB DOC) [file pone.0009425.s002.doc]

**Figure S2**

| **Protein name** | **Reference** |
| --- | --- |
| Pbx1 | (Berkes et al, 2004) |
| PC4 | (Micheli et al, 2005) |
| Id | (Benezra et al, 1990) |
| E12/E47 | (Lassar et al, 1991) |
| BRG1 | (de la Serna et al, 2001) |
| Prohibitin | (Sun et al, 2004) |
| HP1β | (Yahi et al, 2008) |
| PRMT5 | (Dacwag et al, 2007) |

**References**

Benezra R, Davis RL, Lockshon D, Turner DL, Weintraub H (1990) The protein Id: a negative regulator of Helix-Loop-Helix DNA binding proteins. *Cell* **61:** 49-59

Berkes CA, Bergstrom DA, Penn BH, Seaver KJ, Knoepfler PS, Tapscott SJ (2004) Pbx marks genes for activation by MyoD indicating a role for a homeodomain protein in establishing myogenic potential. *Mol Cell* **14**(4)**:** 465-477

Dacwag CS, Ohkawa Y, Pal S, Sif S, Imbalzano AN (2007) The protein arginine methyltransferase Prmt5 is required for myogenesis because it facilitates ATP-dependent chromatin remodeling. *Mol Cell Biol* **27**(1)**:** 384-394

de la Serna IL, Carlson KA, Imbalzano AN (2001) Mammalian SWI/SNF complexes promote MyoD-mediated muscle differentiation. *Nat Genet* **27**(2)**:** 187-190

Lassar AB, Davis RL, Wright WE, Kadesch T, Murre C, Voronova A, Baltimore D, Weintraub H (1991) Functional activity of myogenic HLH proteins requires hetero-oligomerization with E12/E47-like proteins in vivo. *Cell* **66**(2)**:** 305-315

Micheli L, Leonardi L, Conti F, Buanne P, Canu N, Caruso M, Tirone F (2005) PC4 coactivates MyoD by relieving the histone deacetylase 4-mediated inhibition of myocyte enhancer factor 2C. *Mol Cell Biol* **25**(6)**:** 2242-2259

Sun L, Liu L, Yang XJ, Wu Z (2004) Akt binds prohibitin 2 and relieves its repression of MyoD and muscle differentiation. *J Cell Sci* **117**(Pt 14)**:** 3021-3029

Yahi H, Fritsch L, Philipot O, Guasconi V, Souidi M, Robin P, Polesskaya A, Losson R, Harel-Bellan A, Ait-Si-Ali S (2008) Differential cooperation between heterochromatin protein HP1 isoforms and MyoD in myoblasts. *J Biol Chem* **283**(35)**:** 23692-23700.
